# Cost of the medical management and prescription pattern for primary open angle glaucoma (POAG) in Ghana–a retrospective cross-sectional study from three referral facilities

**DOI:** 10.1186/s12913-016-1528-x

**Published:** 2016-07-19

**Authors:** Stephen Ocansey, Samuel Kyei, Ama Diafo, Kwabena Nkansah Darfor, Samuel Bert Boadi-Kusi, Peter B. Aglobitse

**Affiliations:** Department of Vision and Hearing Sciences, Faculty of Science and Technology, Anglia Ruskin University, Cambridge, UK; Department of Optometry, School of Allied Health Sciences, College of Health and Allied Sciences, University of Cape Coast, Cape Coast, Ghana; Department of Economics, Faculty of Social Sciences, College of Humanities and Legal Studies, University of Cape Coast, Cape Coast, Ghana

**Keywords:** Cost, Compliance, Ghana, Glaucoma, Medication

## Abstract

**Background:**

Glaucoma is the leading cause of irreversible blindness globally, and treatment involves considerable cost to stakeholders in healthcare. However, there is infrequent availability of cost information and patterns of management, especially in developing countries. This study determined the cost of the medical management of POAG, adherence, and pattern of medication prescription in Ghana.

**Methods:**

A retrospective cross-sectional study involving 891 Primary Open Angle Glaucoma (POAG) cases seen in the year 2012 at three referral facilities. Demographics, ocular history, resource consumption, medication, test, surgery and other related cost were extracted from 84 patients who had fully complied with their treatment to calculate total cost (TC) based on 2012 estimates. Glaucoma drugs prescribed to patients who had adhered to all their review visits within the period evident from case folders were recorded and analysed for the prescription pattern.

**Results:**

Out of 891 POAG cases seen in 2012, 351(39.4 %) attended all the required review visits, but only 84 (9.4) had fully and continually adhered to all their treatment regimes. They comprised 41(48.8 %) males and 43(51.2 %) females with a mean age of 65 ± 14.8. Majority of the respondents were elderly above 60 year of age (65.5 %). The total estimated cost for the 84 cases in the year was GH¢ 81,237 ($40,619), comprising GH¢ 72,193 ($36,097) direct medication cost and GH¢9,045 ($4,523) direct non-medication cost (surgery and test cost), and an average of GH¢ 967 ($484) for a mean visit of 5.6 ± 1.1 in the year. A total of 673 glaucoma medications had been prescribed for 351 patients for the year, with timolol being the most prescribed (64.19 %) and monotherapy as the most adopted form of therapy (61.06 %). Age and income showed concurrent increase with cost (P ≤ 0.05).

**Conclusions:**

Cost of managing glaucoma constitutes a substantial financial burden and influenced the pattern of medication prescription.

## Background

Glaucoma is a group of optic neuropathies which can lead to progressive and irreversible visual field damage, and is currently the leading cause of irreversible blindness in the world [1]. Risk factors that can predispose one to glaucoma include sustained intraocular pressure (IOP), age, genetics, race, and prolonged use of steroids. Other risk factors are concomitant systemic and ocular conditions such as diabetes and high myopia [1, 2].

Estimates based on published data, in conjunction with prevalence studies have shown that glaucoma is the second leading cause of blindness in the world after cataract, and it is therefore given due public health importance globally [3, 4]. In 2010, it was estimated that 60.5 m people were living with the condition (44.7 m with Primary Open Angle Glaucoma (POAG)), of whom 11.1 m are projected to go blind by 2020 (6.7 m will be blind in both eyes) [5]. In West Africa, the most prevalent type of glaucoma is POAG with 4 % of the population said to be suffering from it [6, 7]. As the risk of POAG increases with age, and with the group of those over 40 years of age constituting more than 25 % of Africa’s population and growing, more people could be living with the condition than reported [6]. Recently, Ghana was ranked as having the second highest rate of glaucoma cases in the world, and highest in West Africa. It is estimated that about 8.5 % of the population aged 40 years and older have glaucoma in Ghana, of whom about 70 % are POAG cases [5, 8, 9].

Surgery is generally considered the standard form of treatment in POAG [2, 6], but in Ghana there is lack of uptake of this option due to inadequate access, high cost, superstition and socio-cultural beliefs which means that many glaucoma patients are treated medically [8–10]. However when the medical option is preferred, the indefinite treatment protocols subject many patients to health care and financial challenges. Moreover, in Ghana the National Health Insurance Scheme (NHIS) established under Act 650 of 2003 by the Government of Ghana is currently facing financial challenges [10, 11]. The NHIS enjoins patients to pay an annual renewable premium to access free health care, including some ophthalmic services [10]. However, since the inception of the NHIS in 2003, hospitals out-patient departments have been inundated with increasing number of patients forcing the government to make cuts to some services and medications, due to delays in paying for the services of health care providers [11]. Consequently, the list of glaucoma medications covered by the NHIS which could be prescribed free under the scheme to glaucoma patients if registered are limited to Acetazolamide (Carbonic Anhydrase Inhibitor), Betaxolol, Pilocarpine (miotics), and Timolol (beta blocker) [11]. Unfortunately, the list does not include more potent medications, such as Latanoprost (prostaglandin analogue) which have been proven to be better at lowering IOP [11–13]. Comparatively, the most effective medications often have higher acquisition cost forcing many patients to rely on the few selected less expensive drugs. In addition, important diagnostic tests such as Visual Field Test (VFT) and Nerve Fiber Layer Assessment (NFLA) are excluded from the NHIS coverage because of the extra burden it will place on public funds. But to many patients, the likelihood of going blind and then not being able to work or perform activities of daily living is far more expensive. Therefore, glaucoma is not only a public health problem but also a developmental problem that must be confronted. However, there is a lack of adequate information about cost and patterns of management and their probable influence on treatment compliance, which has the potential to affect public health planning and clinical practice. This study seeks to estimate the direct costs associated with the medical management of POAG, examine the pattern of glaucoma drug prescription, as well as the adherence to treatment in Ghana.

## Methods

The study was a hospital based retrospective cross-sectional one which involved reviewing all diagnosed glaucoma cases at three eye referral hospitals in the Central Region of Ghana, namely the Cape Coast Teaching, Bishop Ackon Memorial and Our Lady of Grace Hospitals. These hospitals were chosen because they serve as glaucoma referral centers and cater for a large number of patients in the Western and Central coastal parts of Ghana and beyond. Apart from them serving as tertiary eye referral centers, they also have the full range of eye care facilities and eye care personnel including, Ophthalmic Nurses, Optometrists and Ophthalmologists who diagnose and manage glaucoma cases seen at these facilities. An initial study period of 2010–2014 was considered for data collection, but due to the peculiar problem of the limited number of Ophthalmologist in Ghana [14], there were frequent periods within the initially chosen period when Ophthalmologists were not continually present to see glaucoma at the three facilities. To ensure that POAG cases in the study had been on therapy for a least a year, were diagnosed by an Ophthalmologist according to the International Classification of Diseases (Ninth Revision, Clinical Modification 365.11; 365.12; 365.04), and were always reviewed by an Ophthalmologist on their subsequent visits, only the year 2012 was selected because it was the only year during which Ophthalmologists were concurrently present at the three facilities throughout the year. Protocols for diagnosed POAG were similar for the three centers, and involved persistent high IOP, slight lamp examination of anterior chamber angle (gonioscopy was not done in all cases), and VFTs. All medications prescribed and test conducted were all recorded in the patients’ folders.

Patients’ folders were assessed for information on demographic background, medical and ocular history, ophthalmologist visits, medications prescribed, surgical procedures, and clinical tests such as VFT, IOP measurement and refractive assessment which were manually extracted from the folders and entered into excel spread sheet by Optometrists who are familiar with this procedure. Information on prices of few medications which were unavailable and were obtained outside the hospital pharmacies and tests conducted at private facilities were acquired through telephone calls to the appropriate pharmacies and facilities involved.

In Ghana, as part of glaucoma treatment protocol, patients are required to report back to the treatment centers either monthly or every three months for their cases to reviewed or monitored. Consequently, it was possible to access compliance rate for review visits and medications prescribed for the selected period of one year. Those included in the cost evaluation were above 18 years, had been on continuous treatment for at least the selected year of 2012, and had fully adhered to all medications and tests prescribed for them. Due to the importance of treatment compliance in managing POAG, attending Ophthalmologist always inquired from patients about usage of previously prescribed medications as part of the review protocol, and the patient responses including all tests are duly recorded in their folders. This inclusion criterion allowed us to determine full treatment compliance rate, and also guaranteed that the cost evaluation was robust and free from lost information due to failure to use medications or undergo tests as prescribed for glaucoma since such test results are also filed together with case folders. Those included in the prescription pattern analysis involved patients who had made all their required scheduled reviews, and therefore had the folders containing medications prescribed to cover the selected year. Patients’ folders which did not meet these requirements were excluded due to the possible lost information for periods they missed their visits. Cases of Angle Closure Glaucoma (ACG) and secondary glaucoma were excluded because of unrelated expenses [2, 15].

After data on ocular drugs prescribed, each test, and each resource consumed was collected for each visit during the study period, an average cost for each visit was derived as the unit cost of each patient. The average cost for each visit was then used to estimate the direct costs associated with each visit as done in other studies [14, 16–18]. Total costs (TC) per year (PY) constituted the direct medication cost and direct non-medical cost associated with surgery, and tests such as VFT, NFLA, IOP and refraction (of which the glaucoma was the cause) using an average exchange rate (2012) GH¢2 = $1) [16]. Then an average TC per year was calculated for the entire sample by dividing the TC by the total number of cases. Thus, the average TC was adjusted for the total number of visits to achieve a unique TC for each patient. In order to determine the factors that influenced the cost of glaucoma, the authors regressed the sample’s *TC* (which was a weighted average of TC weighted by the total number of visits to the health facility for each patient) on some selected socioeconomic and other factors (P ≤ 0.05). The model used was specified as

$$ TC={\beta}_0+{\beta}_1 Age+{\beta}_2 Sex+{\beta}_3 Income+{\beta}_4 Educ+{\beta}_5 drugtyp{e}_i+{\varepsilon}_i $$

Where,

*Age* was age of respondents in years, *Sex* was the sex of respondents as either male or female, income was the average income of the patient whiles drug type was the anti-glaucoma medication used by the patient in management of glaucoma. This variable was broken into the various types of medication with *Timolol* as the base. *ε*_*i*_ is the stochastic disturbance term. In order to present a robust result, the authors corrected for heteroscedasticity, by making use of robust standard errors. All data were analysed using the Statistical Package for Social Sciences (SPSS) 16 and Stata 11.2 which was used for the linear regression analysis.

The study adhered to the Helsinki Declaration on Research involving human subjects. Anonymity of patients whose folders were reviewed was ensured at all times. This study was reviewed and approved by the Department of Optometry, University of Cape Coast and the Eye facilities involved.

## Results

A total of 891 diagnosed POAG cases were reviewed for the selected year of 2012. Out of that total POAG cases, a significant low proportion 351 (39.4 %) made all their required review visits, but only 84 had fully adhered to their treatment regimens. That is, overall only 9.4 % made all required visits, undertook all tests, and confirmed use of all medications prescribed for them as recorded in their folders. Only 6 (7 %) had undergone surgery. Among the 84 who had fully complied with their treatment, 41 (49 %) were male and 43 (51 %) were female. The female proportion among those who complied with their treatment was slightly lower than to the number of female cases 418 (54 %) initially counted but high compared to the male 473 (46 %). More than half (59.4 %) were registered with the NHIS. Thirty six (42.9 %) were aged 70 years and above and all registered by the NHIS. Fifty five (65.5 %) were above the retirement age (in Ghana) of 60 years, of whom 52 (61.9 %) were not involved in any economic activity. More than a quarter of them had no formal education 25 (29.8 %) (Table 1). Patients on average visited the facilities 6 times. Overall, the cost of medication prescribed and tests done and recorded in the folders of 84 patients who had fully adhered to their treatment was calculated, the pattern of medications prescribed to the 351 patients who had made all the required visits were analysed.

**Table 1:** Distribution of estimated cost among respondents

Demographics	Male	Female	Total cost (Ghc^b^)
Age (years)			
≤30	0 (0^a^)	2 (4.7)	1950 (2.4)
31–40	1 (2.4)	1 (2.3)	1950 (2.4)
41–50	9 (22.0)	4 (9.3)	12592 (15.5)
51–60	6 (14.6)	6 (14.0)	11617 (14.30
61–70	10 (24.4)	9 (20.9)	18360 (22.6)
70+	15 (36.6)	21 (48.8)	34769 (42.8)
Educational level			
No formal education	4 (9.8)	21 (48.8)	24127 (29.7)
Primary/Basic	12 (29.3)	12 (27.9)	23234 (28.6)
Secondary/Technical/	9 (22.0)	4 (9.3)	12592 (15.5)
Vocational			
Post-secondary/Tertiary	16 (39.0)	6 (14.0)	21284 (26.2)
Income (Ghc^b^)			
0–599	31 (75.6)	42 (97.7)	70676 (87.0)
600–999	6 (14.6)	4 (9.8)	5687 (7.0)
>1000	4 (9.8)	1 (2.3)	4874 (6.0)
Total	*N* = 41 (100)	*N* = 43 (100)	81237 (100)

^a^percentages are in parenthesis ^b^Ghana cedi (2Gh¢ = $1 in 2012 in nominal terms)

The estimated total cost (PY) for the 84 fully compliant patients was calculated to be, total medication cost = GH¢ 72,193 ($36,087) + total non-medication cost = GH¢9,045 ($4,523) = GH¢81,237 ($40.619). An average cost case per year was determined to be = GH¢ GH¢ 967 ($484). Assuming if all the POAG 891 cases had fully adhered to their treatment regime, and followed a similar course of treatment, the total estimated cost (PY) could have been GH¢861,597 ($430,799).

The results (Table 2) indicated that sex and educational status of the patients were not statistically significant in accounting for the variations in total estimated cost. The regression results showed that the model was a good fit with an R^2^ of 34 % indicating that 34 % of the variations in total estimated cost are accounted for by the regression. The F-statistics also showed that at less than five percent level of significance, the independent variables were jointly significant in accounting for the variations in total calculated cost. The model also corrected for the problem of heroscedasticity by making use of robust standard errors. Age was found to be important in influencing the variations in total estimated cost such that a unit increase in age will lead to an increase in total estimated cost by 0.43. Income was also found to be important in influencing the total estimated cost. A unit increase in income leads to a 0.01 increase in total estimated cost. The size of the coefficient of income seemed to suggest that it was not so important in terms of the margin of increase that is occasioned on total calculated cost. The type of drug prescribed was also found to be important in influencing total estimated cost. From the regression output, total estimated cost was influenced more by Xalatan relative to the other medications. From the table, a unit increase in the purchase of Xalatan will increase total cost by 12.03 units. Of all the medications included in the model, Xalatan was the most expensive, and therefore it could be predicted that, if it was prescribed and used more often by the patients relative to the other medications, the total calculated cost would be expected to increase.

**Table 2:** Relationship between estimated cost and selected independent variables

Total cost	Coef.	Robust Std. Err	t	P > |t|	[95 % Conf. interval]	
Sex	−1.65	3.88	−0.43	0.672	−9.38	6.08
EduStatus						
Basic Edu.	5.65	4.02	1.40	0.165	−2.38	13.67
Secondary Edu.	1.85	4.28	0.43	0.667	−6.68	10.38
Higher Edu.	−2.92	5.79	−0.5	0.616	−14.47	8.63
Age	0.43	0.10	4.03	0.000^a^	−0.65	0.22
Income	0.01	0.00	2.45	0.017^a^	0.00	0.020
DrugType						
Cusimolol	1.22	7.86	0.16	0.877	−14.44	16.88
Optimol	4.43	6.62	0.67	0.505	−8.76	17.62
Xalatan	12.02	5.64	2.13	0.036^a^	0.78	23.26
Prostan	7.21	5.68	1.27	0.209	−4.13	18.54
Others	3.74	4.12	0.91	0.367	−4.47	11.96
_cons	32.12	12.73	2.52	0.014	6.74	57.49

^a^= Indicates significance at 5 %

*Number of observations =84. Overall effect* [F (11, 72) = 10.85, *p* = 0.000]

**Fig. 1:**
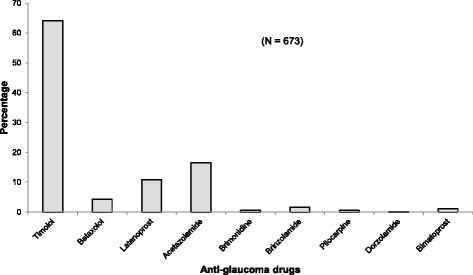
Distribution of anti-glaucoma medication prescribed by trade name

**Fig. 2:**
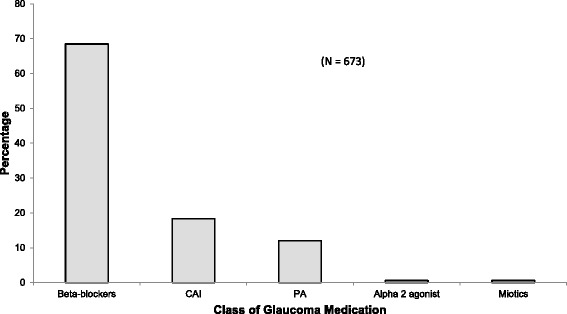
Distribution of class of anti-glaucoma medications prescribed. *CIA: Carbonic Anhydrase Inhibitors PA: Prostaglandin* analogs

Of the 351 cases who made all the required visits for the year, a total of 673 anti-glaucoma medications had been prescribed. The medications were grouped according to the active ingredient in them. Timolol was the most prescribed anti-glaucoma medication, representing 432 (64.19 %) while Dorzolamide was the least prescribed 1 (0.2 %) (Fig. 1). This translated into beta-blockers being the most frequently prescribed class of drugs (Fig. 2). Monotherapy (use of single medication) was also the most used form of therapy in 287(61.06 %) cases, followed by two drug combination 163 (34.68 %) (not single vials), and then three drug combinations 20 (4.26 %) (Table 3).

**Table 3:** Distribution of anti-glaucoma medications prescribed for 351 patients who attended all reviews for one year

Form of therapy	Number of drugs prescribed	Percent (%)
1. Monotherapy		
Timolol	257	89.6
Betaxolol	21	7.3
Latanoprost	8	2.7
Acetazolamide	1	0.4
2. Two drug combination^a^		
Tim + Ace	96	59.6
Bet + Ace	5	3.3
Bim + Lat	51	31.6
Tim + Brimo	4	2.5
Tim + Dorzo	1	0.6
Tim + Bim	1	0.6
Bet + Lat	3	1.8
3. Three drug Combination		
Tim + Lat + Brim	4	20
Tim + Bim + Brin	7	35
Tim + Ace + Lat	7	35
Tim + Ace + Pil	2	10
Overall Total	673^b^	100

^a^Combinations are not single vials ^b^Represents individual medication counts

*Tim* timolol, *Ace* acetazolamide, *Bim* bimatoprost, *Lat* latanoprost, *Brimo* brimonidine, *Dorzo* dorzolamide

## Discussion

This study found that a substantial proportion of patients (65 %) did not adhere to their scheduled review visits, and an overall low full adherence rate of less than 10 %. This confirms the well-known finding that a significant number of patients fail to fully comply with their review visits and glaucoma medication precisely as prescribed. Moreover, it is likely that the non-adherence rate might even be higher than estimated, if all cases of POAG had been included. By proportion, the slightly more female than male found in the sample may not directly be related to the diagnosed glaucoma, as sex did not influence the estimated cost. Some studies have suggested that sex is not linked to POAG, while others found an association [5, 19–21]. In this study, the slightly higher number of female than male in this study may be due to the higher proportion of female in the Ghana population and not an associated with POAG cases [22].

Glaucoma medications were found to be the major cost drivers to treatment as there were only few patients who had had surgery. The estimated average cost of GH¢ 967 ($484) per POAG patient per year is high considering that majority 61.9 % of the patients were less economically active and were not involved in any income generating activities. In 2012, the minimum monthly wage was 120.96 ($60) and with only 28.4 % of the Ghana population who were below the international poverty line of US$1.25 per day (2007–2011), the cost to the individual could be considered high [23]. There are recently reported financial challenges facing the Ghana NHIS therefore the projected TC of GH¢861,597 ($430,799) per year (assumed full adherence rate for all 891 patients) can be seen as additional burden for a country with Gross National Income (GNI) per capita (2012) of $1550 [22, 23]. On the other hand, the estimated TC could even be higher considering that costs related to eye care facilities use, equipment use, consultations and public health programs which were not part the cost evaluation. Additionally, other intangible costs such as stress, loss of leisure time, failure to participate in societal activities, modification of social and economic decisions were not factored in the calculation.

The indefinite management of glaucoma might have influenced the high proportion of NHIS registration among the patients. The national registration rate for the NHIS is reported to be around 70 % according to Government figures, but a study have suggested the number could be far lower [22]. The study revealed that, as many as twice high income earners are registered unto the scheme compared to the poor because of their ability to pay an annual renewable premium [22]. In this study, patients who were found not be registered with the NHIS could be attributed to their inability to afford the cost of registration or renewal fee, and this can possibly be cited to partly account for the gap in adherence rate. Indeed, all individuals who were aged 70 years and above were on the scheme because they were granted free registration by law.

The study showed a significant relationship between age and total estimated cost. Glaucoma is a chronic and progressive condition that is asymptomatic in its early stages, resulting in delayed case reporting. In Ghana, access to eye care is also a challenge to many and therefore patients might seek care at the advanced stages when visual defects become manifest [10]. At more advanced stages of the condition, there is likely to be an increase in reporting time, more resource consumption, and shift to more expensive medications to slow progression [12, 13]. The increase in reporting time could also be attributed to certain factors such as the elderly having access to some welfare packages such the free NHIS registration that enabled them increase the number of hospital visits. The association of cost with income may also reflect the unequal socio-economic status in Ghana, and the link between the purchasing power (ability to afford) and income.

The pattern of prescription found in the study may also underline cost implications and prescriber preferences. The predominance reliance on monotherapy and particularly the use of Timolol is uncommon in similar studies [12, 24]. In this study, this can be attributed to a preference for low-priced medication, its availability on the NHIS medication list, and prescriber preference which perhaps is due to the uncomplicated nature of administering it alone or in combination with other class of glaucoma medications [14, 25–28]. Though Acetazolamide has some reported systemic side effects mainly because of its oral route of administration [24], it was relatively prescribed for short-term use in this study, probably motivated by the desire to reduce IOP since the patients could not afford more expensive medications which has IOP-lowering potency. It is also the least expensive medication on the NHIS list and may underline the higher prescription rate. Other studies have reported it to be one of the least prescribed [29]. On the contrary, though Latanoprost is known for its potency for reducing IOP [13, 15], it was less frequently prescribed because of its high acquisition cost.

While the findings from this retrospective study are consistent with results from similar glaucoma cost estimation studies, a number of methodological limitations may limit the extrapolation of the data to a national scale. First, the low rate of adherence among the study population affected the sample size, and for that matter the total cost estimation. The costing evaluation method employed may also have led to underestimation of the TC because it did not include indirect cost associated POAG treatment such as facility use, diagnostic, personnel as well as indirect cost related to transportation for the patients and escorts. It is also possible some information may have been lost due to tracking, since the study involved case audit analysis to extract the relevant information from the patient folders.

## Conclusions

This study has a number of clinical implications for treating glaucoma patients and financing care. Though the low full adherence rate found in this study is not all unexpected in the study population regarding medical care, the non-compliance to glaucoma medication maybe detrimental to lowering IOP to slow or halt vision loss. It is reasonable to imply that the high non-adherence rate is likely to have led to delayed and unsuccessful management. Therefore, it is important to find clinical ways to improve adherence among POAG patient when the medical treatment option is preferred. It has long been suggested that daily dose frequency, forgetfulness, inconvenience and side effects account for non-adherence among glaucoma patients [30]. This study found that there were cases of multiple drug prescription that required frequent dosage which many patients may find be problematic. For such patients, single potent drugs that do the work of more than one drug, as well as drugs that need to be taken as few times a day as possible would likely enhance compliance. Further, clinicians need to give clear instructions because inadequate communication by practitioners could have influenced the compliance rate and minimised successful clinical outcomes [31].

The high prevalence of POAG cases in the Ghanaian population coupled with the chronic nature of the condition constitutes financial burden to the individual, and the Government through the NHIS as the largest provider of public health in Ghana. However, it is obvious from the study that the prescription pattern of prescribers were influenced, perhaps partly by the cost of the drugs. There were poor compliance and adherence to treatment schedule with cost as the main factor. Notwithstanding the underlying cost, the likelihood of treatment success must be considered the main factor in choosing the appropriate drugs for a given patient. The inclusion of unbranded prostaglandin analogues in the NHIS drug list, and including essential glaucoma diagnostic tests (such as VFT and NFLA) for those aged 40 years and above in the NHIS may improve adherence. Moreover, considering the emerging aging population in Ghana, the failure to carefully estimate cost implications of treating glaucoma on a National scale has prospects to undermine eye health and derail the NHIS. This study can be considered as exploratory, but the findings highlight cost as a major factor in treating glaucoma patients in Ghana. A more holistic approach that avoids the underlined limitation will go a long way to safeguard the visual health of the Ghanaian populace.

## Abbreviations

ACG, angle closure glaucoma (ACG); GNI, gross national income (GNI); IOP, intraocular pressure (IOP); NFLA, nerve fiber layer assessment (NFLA); NHIS, National Health Insurance Scheme (NHIS); POAG, primary open angle glaucoma; SPSS, statistical package for social sciences (SPSS); TC, total cost; VFT, visual field test.
